# Limited angle tomography for transmission X-ray microscopy using deep learning

**DOI:** 10.1107/S160057752000017X

**Published:** 2020-02-13

**Authors:** Yixing Huang, Shengxiang Wang, Yong Guan, Andreas Maier

**Affiliations:** a Pattern Recognition Lab, Friedrich-Alexander-Universität Erlangen-Nürnberg, 91058 Erlangen, Germany; b Spallation Neutron Source Science Center, Dongguan, Guangdong 523803, People’s Republic of China; c Institute of High Energy Physics, Chinese Academy of Sciences, Beijing 100049, People’s Republic of China; d National Synchrotron Radiation Laboratory, University of Science and Technology of China, Hefei, Anhui 230026, People’s Republic of China; e Erlangen Graduate School in Advanced Optical Technologies (SAOT), 91052 Erlangen, Germany

**Keywords:** transmission X-ray microscopy, deep learning, limited angle tomography

## Abstract

In transmission X-ray microscopy (TXM) systems, the rotation of a scanned sample might be restricted to a limited angular range to avoid collision with other system parts or high attenuation at certain tilting angles. Image reconstruction from such limited angle data suffers from artifacts because of missing data. In this work, deep learning is applied to limited angle reconstruction in TXMs for the first time. With the challenge to obtain sufficient real data for training, training a deep neural network from synthetic data is investigated. In particular, U-Net, the state-of-the-art neural network in biomedical imaging, is trained from synthetic ellipsoid data and multi-category data to reduce artifacts in filtered back-projection (FBP) reconstruction images. The proposed method is evaluated on synthetic data and real scanned chlorella data in 100° limited angle tomography. For synthetic test data, U-Net significantly reduces the root-mean-square error (RMSE) from 2.55 × 10^−3^ µm^−1^ in the FBP reconstruction to 1.21 × 10^−3^ µm^−1^ in the U-Net reconstruction and also improves the structural similarity (SSIM) index from 0.625 to 0.920. With penalized weighted least-square denoising of measured projections, the RMSE and SSIM are further improved to 1.16 × 10^−3^ µm^−1^ and 0.932, respectively. For real test data, the proposed method remarkably improves the 3D visualization of the subcellular structures in the chlorella cell, which indicates its important value for nanoscale imaging in biology, nanoscience and materials science.

## Abstract 2

A deep-learning method for limited angle tomography in synchrotron radiation transmission X-ray microscopies and a demonstration of its application in 3D visualization of a chlorella cell.

## 1. Introduction   

Transmission X-ray microscopy (TXM) has become a very powerful technology for nanoscale imaging in various fields (Wang *et al.*, 2000 ▸, 2016 ▸; Chao *et al.*, 2005 ▸; Sakdinawat & Attwood, 2010 ▸), including materials science (Andrews *et al.*, 2011 ▸; Nelson *et al.*, 2012 ▸), chemistry (de Smit *et al.*, 2008 ▸; Wang *et al.*, 2015*a* ▸) and biology (Shapiro *et al.*, 2005 ▸; Wang *et al.*, 2015*b* ▸). With projection images acquired in a series of rotational angles, tomographic images can be reconstructed according to computed tomography (CT) technologies for 3D visualization of scanned samples. In such applications, TXM is also called X-ray nano-CT (Shearing *et al.*, 2011 ▸; Brisard *et al.*, 2012 ▸; Liu *et al.*, 2018 ▸). A TXM system typically consists of a central stop, a condenser, a sample holder, an objective zone plate and a CCD detector, with X-rays generated from synchrotron radiation or a high-end X-ray source. TXMs typically utilize a pin as the sample holder (Holler *et al.*, 2017 ▸), *e.g.* tip versions for pillar samples, glass capillaries for powder samples, copper capillaries for high-pressure cryogenic samples and grids for flat samples. For tips and capillaries, rotating a sample in a sufficient angular range is not a problem. However, for grids, collision between the grid and the zone plate, which is very near to the rotation axis in TXM systems, might happen at large scan angles. In addition, for flat samples, the lengths of X-rays through the sample increase rapidly at high tilting angles (Barnard *et al.*, 1992 ▸; Koster *et al.*, 1997 ▸), which introduces a high level of scattering and reduces image contrast. Therefore, in these situations, the problem of limited angle tomography arises.

Limited angle tomography is a severely ill-posed inverse problem (Davison, 1983 ▸; Louis, 1986 ▸; Natterer, 1986 ▸; Quinto, 2006 ▸). Using microlocal analysis, edges that are tangent to available X-rays can be well reconstructed while those whose singularities are not perpendicular to any X-ray lines cannot be reconstructed stably (Quinto, 1993 ▸, 2006 ▸). So far, many algorithms have been developed to deal with this task. Among these algorithms, extrapolating missing data is the most straightforward way for limited angle tomography. The iterative Gerchberg–Papoulis extrapolation algorithm (Gerchberg, 1974 ▸; Papoulis, 1975 ▸) based on band-limitation properties of imaged objects has been demonstrated beneficial for improving image quality of limited angle tomography (Defrise & de Mol, 1983 ▸; Qu *et al.*, 2008 ▸; Qu & Jiang, 2009 ▸; Huang *et al.*, 2018*b* ▸). In addition, data-consistency conditions, *e.g.* the Helgason–Ludwig consistency conditions (Helgason, 1965 ▸; Ludwig, 1966 ▸), provide redundancy and constraint information of projection data, which effectively improves the quality of extrapolation (Louis & Törnig, 1980 ▸; Louis, 1981 ▸; Prince & Willsky, 1990 ▸; Kudo & Saito, 1991 ▸; Huang *et al.*, 2017 ▸). Nevertheless, such extrapolation methods have only achieved limited performance on real data, which typically contain complex structures and are very difficult to extrapolate.

Iterative reconstruction using sparse regularization technologies, particularly total variation (TV), has been widely applied to image reconstruction from insufficient data. TV methods employ the sparsity information of image gradients as a regularization term. Therefore, noise and artifacts, which tend to increase the TV value, can be reduced via such regularization. For limited angle tomography, algorithms of adaptive steepest descent projection onto convex sets (ASD-POCS) (Sidky *et al.*, 2006 ▸; Sidky & Pan, 2008 ▸), improved total variation (iTV) (Ritschl *et al.*, 2011 ▸), anisotropic total variation (aTV) (Chen *et al.*, 2013 ▸), reweighted total variation (wTV) (Huang *et al.*, 2016*a* ▸, 2016*b* ▸) and scale-space anisotropic total variation (ssaTV) (Huang *et al.*, 2018*a* ▸) have been proposed. While TV methods achieve good reconstruction results when the missing angular range is small, they fail to reduce severe artifacts when a large angular range is missing. Moreover, they also require expensive computation and tend to lose high-resolution details.

Recently, machine-learning techniques have achieved overwhelming success in a large range of fields including X-ray imaging. In the application of limited angle tomography, pixel-by-pixel artifact prediction using traditional machine learning is one direction (Huang *et al.*, 2019*a* ▸). However, new artifacts might be introduced. Instead, deep-learning methods have achieved impressive results. Würfl *et al.* (2016 ▸, 2018 ▸) proposed to learn certain weights based on known filtered back-projection (FBP) operators (Maier *et al.*, 2019 ▸) to compensate missing data in limited angle tomography. Gu & Ye (2017 ▸) proposed to learn artifacts from streaky images in a multi-scale wavelet domain using the U-Net architecture (Ronneberger *et al.*, 2015 ▸; Falk *et al.*, 2019 ▸). Bubba *et al.* (2019 ▸) utilized an iterative shearlet transform algorithm to reconstruct visible singularities of an imaged object and a U-Net based neural network with dense blocks to predict invisible singularities. In our previous work, we have demonstrated that deep learning is not robust to noise and adversarial examples (Huang *et al.*, 2018*c* ▸). To improve image quality, a data consistent reconstruction method (Huang *et al.*, 2019*b* ▸) is proposed, where deep-learning reconstruction is used as prior to provide information of missing data while conventional iterative reconstruction is applied to make deep-learning reconstruction consistent to measured projection data.

In this work, deep learning is applied to limited angle reconstruction in the field of TXMs for the first time, to the best of our knowledge. Furthermore, training data is vital for deep-learning methods. Without access to real training data, in this work we will investigate the performance of deep learning trained from synthetic data.

## 2. Materials and method   

The proposed limited angle reconstruction method for TXMs consists of two steps: FBP preliminary reconstruction and deep-learning reconstruction as post-processing.

### 2.1. FBP preliminary reconstruction   

For TXM systems with synchrotron radiation, parallel-beam X-rays are used. Each X-ray measures a line integral of the linear attenuation coefficients of a scanned sample, represented as
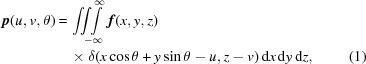
where θ is the rotation angle of the sample, the rotation axis is parallel with the *z* axis, *u* and *v* are the horizontal and vertical position indices at the detector, respectively, ***p***(*u*, *v*, θ) is the log-transformed projection, ***f***(*x*, *y*, *z*) is the attenuation distribution function of the sample, and δ(·) is the Dirac delta function.

In practice, noise always exists in measured projections because of various physical effects, *e.g.* Poisson noise. Since deep-learning methods are sensitive to noise (Huang *et al.*, 2018*c* ▸), noise reduction in input images is preferred. For this purpose, a penalized weighted least-square (PWLS) approach is utilized in projection domain. The objective function for PWLS is as follows (Wang *et al.*, 2006 ▸),
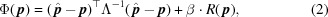
where ***p*** is the vector of the ideal log-transformed projection, 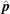 is the vector of the measured log-transformed projection containing noise, ***p***_*i*_ is the *i*th element of ***p***, Λ is a diagonal matrix with the *i*th element equal to an estimate of the variance of 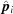, *R*(***p***) is a regularization term and β is a relaxation parameter. The regularization term *R*(***p***) is chosen as
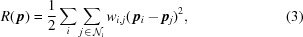
where 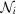 is the four-connectivity neighborhood of the *i*th pixel and the weight *w*_*i*, *j*_ is defined as
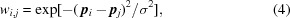
with σ a predefined parameter to control the weight.

The denoised projection is denoted by ***p***′(*u*, *v*, θ). For image reconstruction, the FBP algorithm with the Ram–Lak kernel ***h***(*u*) is applied,
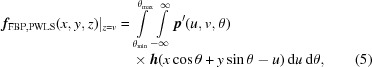
where 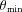 and 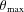 are the start rotation angle and the end rotation angle, respectively, and ***f***_FBP, PWLS_ is the FBP reconstruction from PWLS processed projection data. We further denote the FBP reconstruction from measured projection data without PWLS by ***f***_FBP_, *i.e.* replacing ***p***′(*u*, *v*, θ) by 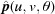 in the above equation.

### 2.2. Deep-learning reconstruction   

#### 2.2.1. Neural network   

The above FBP reconstruction suffers from artifacts, typically in the form of streaks, because of missing data in limited angle tomography. To reduce artifacts, an image-to-image post-processing deep-learning method using U-Net is applied.

**Figure 1:**
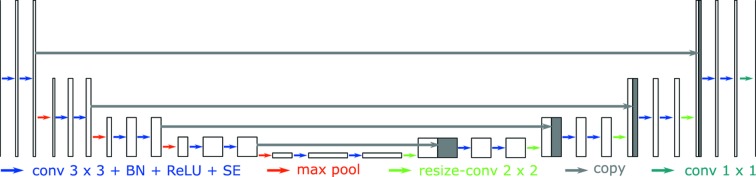
The U-Net architecture for limited angle tomography.

The U-Net architecture for limited angle tomography is displayed in Fig. 1 ▸. The input and output of the U-Net are both 2D images of the same size. Each blue arrow stands for zero-padded 3 × 3 convolution followed by a rectified linear unit (ReLu), a batch normalization (BN) operation, and a squeeze-and-extraction (SE) block (Hu *et al.*, 2018 ▸). Each red arrow represents a max pooling operation to down-sample feature maps by a factor of two. Each green arrow is a bilinear up-sampling operation followed by a 2 × 2 convolution to resize feature maps back. The gray arrows copy features from the left side and concatenate them with the corresponding up-sampled features. The last 1 × 1 convolution operation maps the multi-channel features to a desired output image. Because of the down/up-sampling and copy operations, the U-Net architecture has a large reception field and is able to learn features of multi-scales.

In this work, the input image is a 2D horizontal slice from the FBP reconstruction without or with PWLS pre-processing, *i.e.* ***f***_FBP_ or ***f***_FBP, PWLS_, respectively. The output image is the corresponding artifact image. Hence, a final reconstruction of the U-Net, denoted by ***f***_U-Net_ or ***f***_U-Net, PWLS_ for the input image without and with PWLS, respectively, is obtained by subtracting the input image by its corresponding predicted artifact image. For stable training, the input and output images are normalized to the range [−1, 1] using the maximum intensity value of the input images.

Compared with the original U-Net architecture in the work by Ronneberger *et al.* (2015 ▸), the following modifications are made in the above U-Net architecture to improve its performance for limited angle tomography.

(i) Zero-padded convolution. In the original U-Net architecture, unpadded convolution is used and the image size decreases after each convolution. Hence, information near image boundaries is missing in the output image. In this work, the zero-padded convolution is used to preserve image size. Because of this, the cropping operation is no longer necessary for each copy operation.

(ii) Batch normalization. The BN operation normalizes each convolutional layer’s inputs in a mini-batch to a normal distribution with trained mean shift and variance-scaling values. The BN technique allows neural networks to use higher learning rates and be less sensitive to initialization (Ioffe & Szegedy, 2015 ▸). Therefore, it is a standard operation for convolutional neural networks nowadays.

(iii) Squeeze-and-extraction. The SE block (Hu *et al.*, 2018 ▸) squeezes global spatial information into a channel descriptor by first using global average pooling. Afterwards, channel-wise dependencies are captured by a nonlinear excitation mechanism, which emphasizes multi-channel activations instead of single-channel activation. The SE technique adaptively recalibrates channel-wise feature responses to boost the representation power of a neural network.

(iv) Resize and 2 × 2 convolution. The original U-Net architecture uses a deconvolution operation for up-sampling, which introduces checkerboard artifacts (Odena *et al.*, 2016 ▸). To avoid this, we first choose to resize each feature map using bilinear up-sampling with a scaling factor of two. Afterwards, a 2 × 2 convolution operation is applied.

(v) Output and loss function. The original U-Net is proposed for biomedical image segmentation, where the number of segmentation classes decides the channel number of the output image and each channel is a binary vector containing elements of 0 or 1. For segmentation, a softmax function is typically used to determine the highest probability class. Associated with the softmax activation in the output layer, the cross entropy loss function is typically used for training. As mentioned previously, the output image is a one-channel 2D artifact image in this work. Therefore, the result of the 1 × 1 convolution is directly used as the output without any softmax function. Correspondingly, an ℓ_2_ loss function is used for training.

#### 2.2.2. Data preparation   

In order to reconstruct a sample from limited angle data using deep learning, training data is vital. However, on the one hand it is very challenging to obtain a sufficient amount of real data; on the other hand, for most scans only limited angle data are acquired and hence reconstruction from complete data as ground truth is not available. Because of the scarcity of real data, we choose to train the neural network from synthetic data. For this purpose, two kinds of synthetic data are generated.

(i) Ellipsoid phantoms. 3D ellipsoid phantoms are designed with two large ellipsoids to form an outer boundary, two middle-sized ellipsoids to simulate the cup-shaped chloroplast, 20 small ellipsoids to mimic lipid bodies, and 50 high-intensity small-sized ellipsoids to simulate gold nanoparticles which are contained in the sample for geometry and motion calibration (Wang *et al.*, 2019 ▸). The locations, sizes and intensities of the ellipsoids are randomly generated. Since many samples are immobilized in a certain condition, *e.g.* in an ice tube in this work, a background with a constant intensity of 0.002 µm^−1^ is added.

(ii) Multi-category data. For a certain parallel-beam limited angle tomography system, no matter what kinds of objects are imaged, the projections and the FBP reconstructions should follow the mathematics in equations (1) and (5). In addition, based on the theories of transfer learning (Pan & Yang, 2010 ▸) and one/zero-shot learning (Li *et al.*, 2006 ▸; Palatucci *et al.*, 2009 ▸), a neural network trained for one task can also generalize to another similar task. Therefore, in this work, images of multi-categories are collected to train the neural network for complex structures, for example, optical microscopy algae images and medical CT images. Note that although TXMs data for chlorella cells, the test sample in this work, are not accessible, data of algae cells in other imaging modalities, especially in optical microscopies, are abundant. Images in other modalities also share plenty of useful structure information as TXMs do.

### 2.3. Experimental setup   

#### 2.3.1. Synthetic data   

For deep-learning training, 10 ellipsoid phantoms with a size of 512 × 512 × 512 are generated. From each 3D phantom, 20 slices are uniformly selected. From the multi-category data, 400 image slices are collected. Color images are converted to gray intensity images. The above images are further rotated by 90, 180 and 270°. Therefore, 2400 image slices in total are synthesized for training.

**Figure 2:**
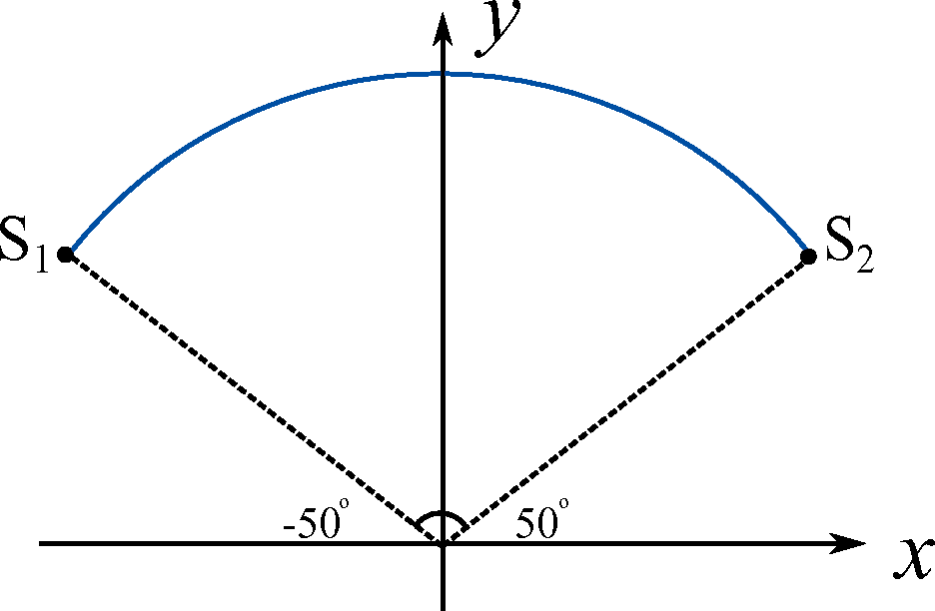
The scanned angular range of the TXM system is from −50° to 50°.

Parallel-beam sinograms are simulated from rotation angles −50° to 50° with an angular step of 1°, as displayed in Fig. 2 ▸. The detector size is 512 with a pixel size of 21.9 nm. To improve the robustness of the neural network to noise, Poisson noise is simulated considering a photon number of 10^4^, 5.0 × 10^4^ or 10^5^ for each X-ray before attenuation. Here, multiple dose levels are used to improve the robustness of the neural network to different levels of noise. For training, 1200 preliminary image slices with a size of 256 × 256 are reconstructed by FBP using the Ram–Lak kernel directly from noisy projection data for the 600 original slices and their 90° rotations, while the other 1200 slices are reconstructed from projection data processed by two iterations of PWLS. To obtain the diagonal matrix Λ in equation (2), the variance of each detector pixel 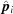 is estimated by the following formula (Wang *et al.*, 2006 ▸),
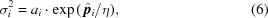
where *a*_*i*_ is set to 0.5 for each pixel *i* and η is set to 1. The value of σ in equation (4) is set to 2.

The U-Net is trained on the above synthetic data using the Adam optimizer for 500 epochs. The learning rate is 10^−3^ for the first 100 epochs and gradually decreases to 10^−5^ for the final epochs. The ℓ_2_-regularization with a parameter of 10^−4^ is applied to avoid large network weights.

For a preliminary quantitative evaluation, the trained U-Net model is evaluated on one new synthetic ellipsoid phantom first. Its limited angle projection data are generated with Poisson noise using a photon number of 10^4^. The projections are denoised by two iterations of PWLS.

#### 2.3.2. Chlorella data   

**Figure 3:**
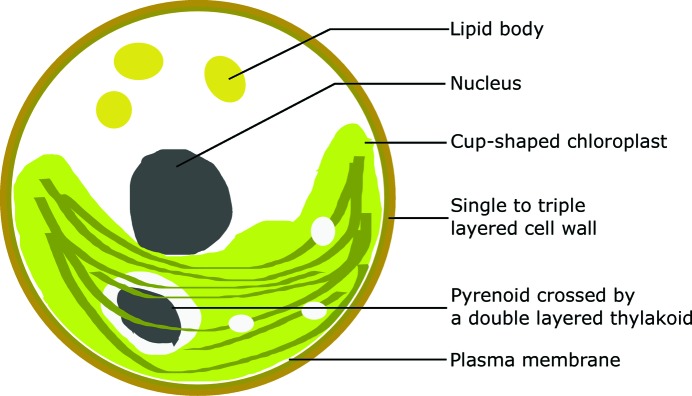
The main structures of a chlorella cell (Baudelet *et al.*, 2017 ▸).

As a demonstration example, a sample of chlorella cells was scanned in a soft X-ray microscope at beamline BL07W (Liu *et al.*, 2018 ▸) in the National Synchrotron Radiation Laboratory in Hefei, China. Chlorella is a genus of single-celled green algae with a size of 2 to 10 µm. It mainly consists of a single- to triple-layered cell wall, a thin plasma membrane, a nucleus, a cup-shaped chloroplast, a pyrenoid and several lipid bodies, as illustrated in Fig. 3 ▸ (Baudelet *et al.*, 2017 ▸).

To hold the chlorella sample, a traditional 100 mesh transmission electron microscopy (TEM) grid was used. Because of the TEM grid, a valid scan of 100° only (−50° to 50° in Fig. 2 ▸ with an angular step of 1°) was acquired to avoid collision between the grid and the zone plate. Rapid freezing of the chlorella sample with liquid nitrogen was performed before scanning to immobilize the cells in an ice tube and suppress the damage of radiation to cellular structures. The X-ray energy used in the experiment was 520 eV for the so-called ‘water window’. Each projection image is rebinned to a size of 512 × 512 with a pixel size of 21.9 nm × 21.9 nm. As the shift of rotation axis (Yang *et al.*, 2015 ▸) and jitter motion (Yu *et al.*, 2018 ▸) are two main causes of image blurriness, they were solved via measurement of geometric moments after acquisition, as described in the work by Wang *et al.* (2019 ▸). The projections are denoised by two iterations of PWLS afterwards.

## 3. Results and discussion   

### 3.1. Ellipsoid phantom results   

**Figure 4:**
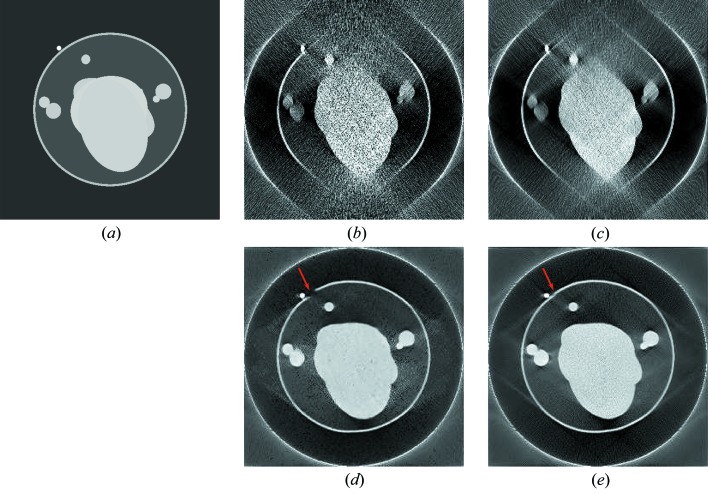
The reconstruction results of the 215th slice in the test ellipsoid phantom without and with PWLS using a photon number of 10^4^, window [0, 0.02] µm^−1^. The boundary indicated by the red arrow is disconnected in ***f***_U-Net_, while it is reconstructed in ***f***_U-Net, PWLS_. The RMSE of each image with respect to the corresponding reference with the unit 10^−3^ µm^−1^ is as follows: (*a*) ***f***_Reference_; (*b*) ***f***_FBP_, 3.61; (*c*) ***f***_FBP, PWLS_, 3.45; (*d*) ***f***_U-Net_, 1.65; and (*e*) ***f***_U-Net, PWLS_, 1.58.

The reconstruction results without and with PWLS for the 250th slice of the test ellipsoid phantom using a photon number of 10^4^ are displayed in Fig. 4 ▸. The root-mean-square error (RMSE) inside the field-of-view (FOV) of each image slice with respect to the corresponding reference slice is displayed in the subcaption. In Figs. 4 ▸(*b*)–4 ▸(*e*), the outer ring is caused by the lateral truncation and it is preserved to mark the FOV.

The FBP reconstruction from 100° limited angle data without PWLS pre-processing, ***f***_FBP_, is displayed in Fig. 4 ▸(*b*). Compared with the reference image ***f***_Reference_, only the structures with an orientation inside the scanned angular range (Fig. 2 ▸) are reconstructed while all other structures are severely distorted. In addition, the Poisson noise pattern is clearly observed because of the low dose. In contrast, Poisson noise is prominently reduced by PWLS in ***f***_FBP, PWLS_, as displayed in Fig. 4 ▸(*c*). The U-Net reconstruction with the input of ***f***_FBP_ is displayed in Fig. 4 ▸(*d*), where most ellipsoid boundaries are restored well. The RMSE inside the FOV is reduced from 3.61 × 10^−3^ µm^−1^ in ***f***_FBP_ to 1.65 × 10^−3^ µm^−1^ in ***f***_U-Net_. This demonstrates the efficacy of deep learning in artifact reduction for limited angle tomography. However, some Poisson noise remains in Fig. 4 ▸(*d*). In particular, the boundary indicated by the red arrow is disconnected in ***f***_U-Net_. The U-Net reconstruction with the input of ***f***_FBP, PWLS_ is displayed in Fig. 4 ▸(*e*), achieving the smallest RMSE value of 1.58 × 10^−3^ µm^−1^. Importantly, the disconnected boundary fragment indicated by the red arrow is reconstructed in ***f***_U-Net, PWLS_. This demonstrates the benefit of PWLS pre-processing.

The average RMSE and structural similarity (SSIM) index of all slices in the FBP and U-Net reconstructions without and with PWLS for the test ellipsoid phantom are displayed in Table 1 ▸. The U-Net reduces the average RMSE value from 2.55 × 10^−3^ µm^−1^ in ***f***_FBP_ to 1.21 × 10^−3^ µm^−1^ in ***f***_U-Net_. With PWLS, the average RMSE is further reduced to 1.16 × 10^−3^ µm^−1^ in ***f***_U-Net, PWLS_. Consistently, ***f***_U-Net, PWLS_ achieves a larger SSIM index than ***f***_U-Net_. This quantitative evaluation also demonstrates the efficacy of the U-Net in limited angle tomography and the benefit of PWLS pre-processing.

### 3.2. Chlorella results   

**Figure 5:**
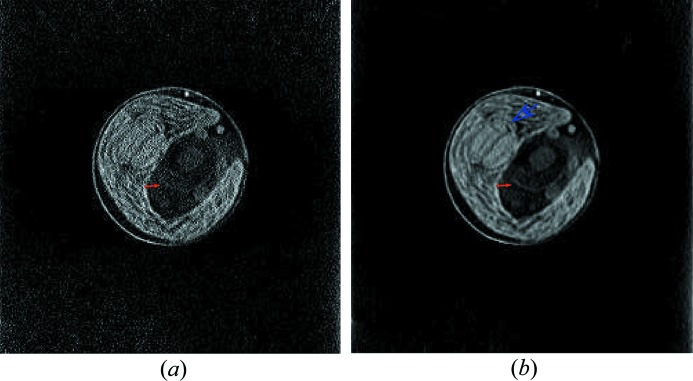
The 103rd slice from the sagittal view reconstructed from projections without and with PWLS pre-processing. The nucleus membrane in (*a*) and (*b*) is indicated by the red solid arrow. The pyrenoid membrane is indicated by the blue hollow arrow. Window: [0, 0.015] µm^−1^. (*a*) ***f***_sag, FBP_ and (*b*) ***f***_sag, FBP, PWLS_.

To demonstrate the benefit of PWLS for the chlorella data, horizontal slices are reconstructed by FBP from the chlorella projection data without or with PWLS processing. A 3D volume is obtained by stacking the horizontal slices. Sagittal slices are obtained by reslicing the volume into 256 slices in the sagittal view. The sagittal slices from projections without and with PWLS are denoted by ***f***_sag, FBP_ and ***f***_sag, FBP, PWLS_, respectively. The results of the 103rd slice are displayed in Fig. 5 ▸. Fig. 5 ▸(*a*) shows that the subcellular structures of cell wall, chloroplast, lipid bodies, nucleus and pyrenoid are reconstructed. However, because of noise, the nucleus membrane is barely seen, which is indicated by the red solid arrow. In contrast, with PWLS, the nucleus membrane is observed better, as indicated by the red solid arrow in Fig. 5 ▸(*b*). Moreover, the textures in the cup-shaped chloroplast are also observed better in Fig. 5 ▸(*b*) than those in Fig. 5 ▸(*a*). For example, the pyrenoid membrane inside the chloroplast is well observed, as indicated by the blue hollow arrow in Fig. 5 ▸(*b*). These observations demonstrate the benefit of PWLS.

**Figure 6:**
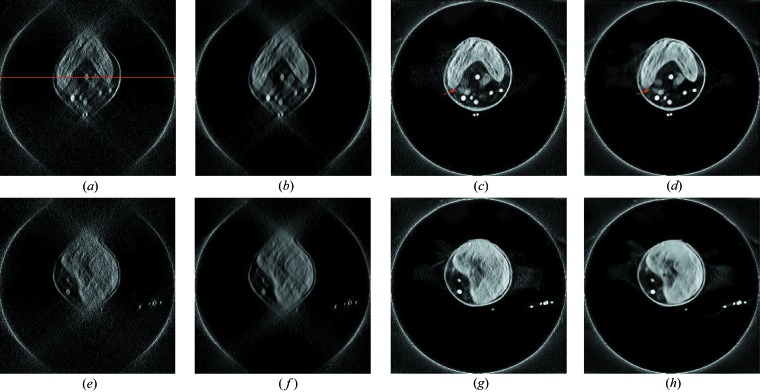
The reconstruction results of two horizontal slices for the chlorella data, window: [0.003, 0.015] µm^−1^. The top row is for the 213th slice while the bottom row is for the 331st slice. The red line in (*a*) indicates the position of intensity profiles in Fig. 7 ▸. (*a*) ***f***_FBP_, (*b*) ***f***_FBP, PWLS_, (*c*) ***f***_U-Net_, (*d*) ***f***_U-Net, PWLS_, (*e*) ***f***_FBP_, (*f*) ***f***_FBP, PWLS_, (*g*) ***f***_U-Net_ and (*h*) ***f***_U-Net, PWLS_.

The reconstruction results of two horizontal example slices are displayed in Fig. 6 ▸. Figs. 6 ▸(*a*) and 6 ▸(*b*) are FBP reconstruction images of the 213th slice without and with PWLS, respectively, where many subcellular structures of the chlorella, *e.g.* the cell wall, chloroplast and lipid bodies, are severely distorted. Compared with Fig. 6 ▸(*a*), Fig. 6 ▸(*b*) contains less noise because of PWLS pre-processing. Their corresponding deep-learning results, ***f***_U-Net_ and ***f***_U-Net, PWLS_, are displayed in Figs. 6 ▸(*c*) and 6 ▸(*d*), respectively. The cell walls are restored and the chloroplasts exhibit a good ‘C’ shape in both images. In addition, the lipid bodies and the gold nanoparticles are well observed. These observations demonstrate the efficacy of deep learning for limited angle tomography on real data. Moreover, the lipid bodies indicated by the arrows in Fig. 6 ▸(*d*) are separated better than those in Fig. 6 ▸(*c*), which highlights the benefit of PWLS pre-processing for deep-learning reconstruction.

For the reconstruction results of the 331st slice displayed in the bottom row, the U-Net is also able to reconstruct the cell wall, the chloroplast and lipid bodies. With PWLS, ***f***_U-Net, PWLS_ in Fig. 6 ▸(*h*) contains less noise than ***f***_U-Net_ in Fig. 6 ▸(*g*), consistently demonstrating the benefit of PWLS.

**Figure 7:**
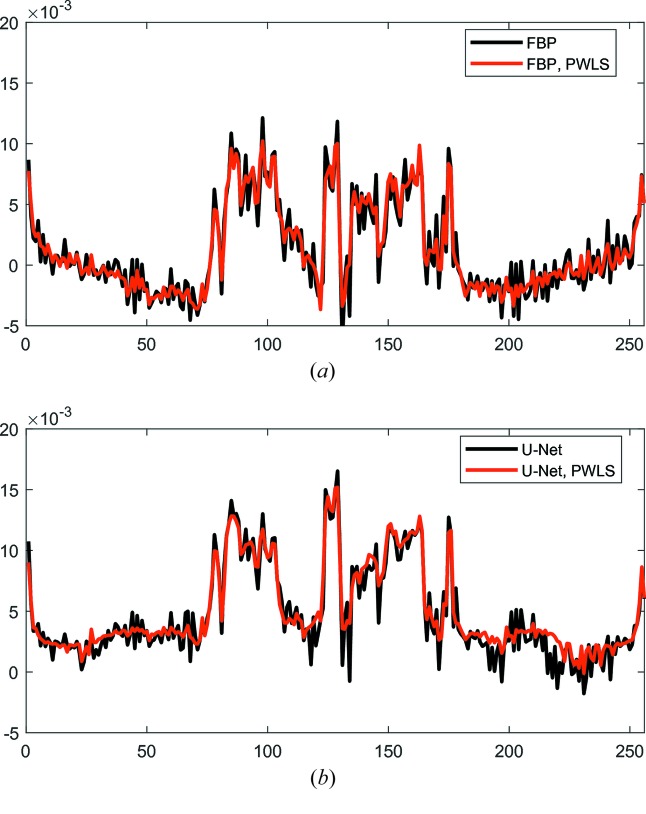
The intensity profiles of a line in the FBP and U-Net reconstructions without and with PWLS. The position of the line is indicated in Fig. 6 ▸(*a*). (*a*) Line profiles of FBP reconstructions and (*b*) line profiles of U-Net reconstructions.

For image-quality quantification, the intensity profiles of a line in the FBP and U-Net reconstructions without and with PWLS are displayed in Fig. 7 ▸. The position of the line is indicated in Fig. 6 ▸(*a*). In Fig. 7 ▸(*a*), the line profiles of ***f***_FBP_ and ***f***_FBP, PWLS_ are displayed. For both profiles, in the pixels of 0–70 and 180–256, the intensity value increases from the center outward, which is a characteristic of cupping artifacts and indicates the existence of data truncation. In the profile of ***f***_FBP_, a lot of high-frequency oscillations are observed, while many of them are mitigated in ***f***_FBP, PWLS_ by PWLS. In Fig. 7 ▸(*b*), high frequency oscillations are observed in the profile of ***f***_U-Net_ as well, while the profile of ***f***_U-Net, PWLS_ has relatively smooth transitions. This demonstrates the benefit of PWLS in avoiding high-frequency noise in the U-Net reconstruction.

**Figure 8:**
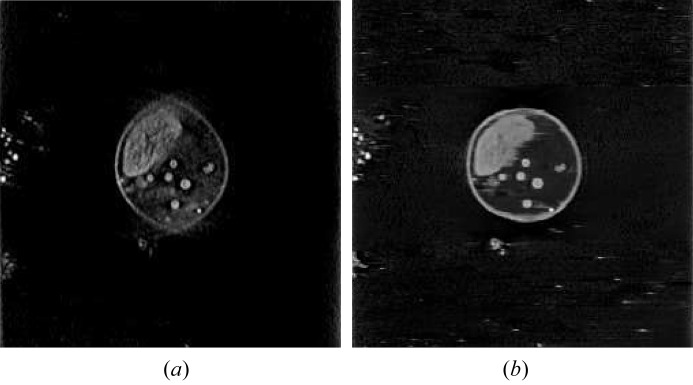
The 150th slices from the sagittal view reconstructed by FBP and U-Net with PWLS pre-processing. Window: [0, 0.015] µm^−1^. (*a*) ***f***_sag, PWLS_ and (*b*) ***f***_sag, U-Net, PWLS_.

In the sagittal view, although structures are observed well for central slices such as the 103rd slice, structures in many other slices are distorted because of missing data. For example, the 150th sagittal slice of the FBP reconstruction ***f***_FBP, PWLS_ is displayed in Fig. 8 ▸(*a*), where the cell wall is severely distorted. With the proposed U-Net reconstruction with PWLS pre-processing, the cell wall is restored in an approximate round shape, as shown in Fig. 8 ▸(*b*).

**Figure 9:**
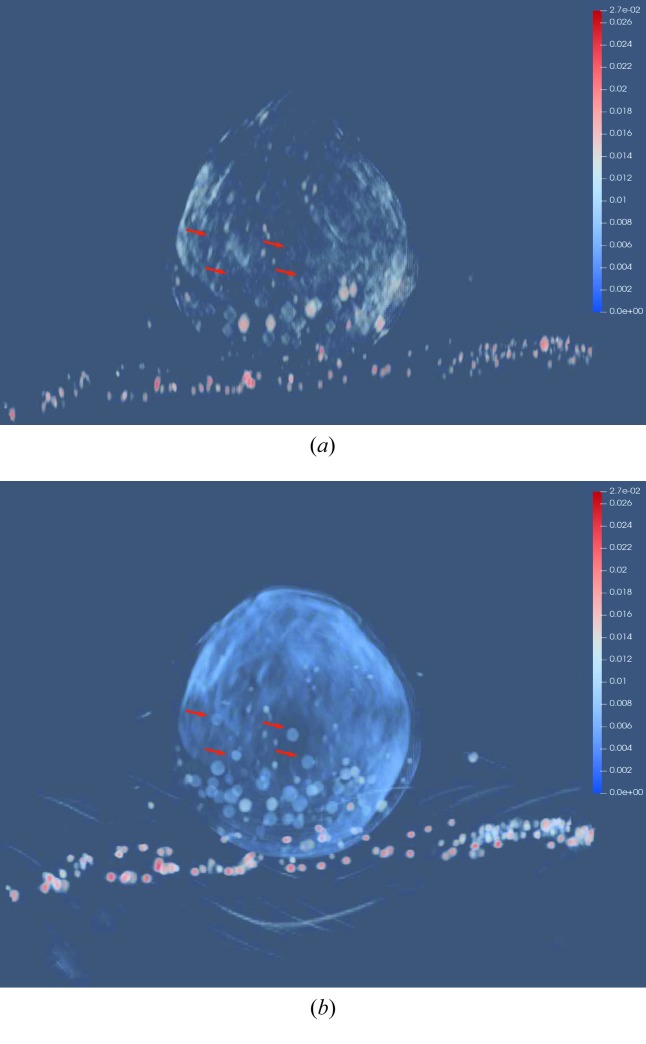
The 3D rendering of the volumes reconstructed by FBP and U-Net with PWLS using the tool of *ParaView*, which is viewed along the *z* direction. The lipids indicated by the arrows are observed well in ***f***_U-Net, PWLS_ while they are barely seen in ***f***_FBP, PWLS_. (*a*) ***f***_FBP, PWLS_ and (*b*) ***f***_U-Net, PWLS_.

The volumes reconstructed by FBP and U-Net with PWLS are rendered by *ParaView*, an open-source 3D visualization tool, and displayed in Figs. 9 ▸(*a*) and 9 ▸(*b*), respectively. Fig. 9 ▸(*a*) shows that the top and bottom parts of the chlorella cell are missing. In addition, the shapes of lipid bodies are distorted. Instead, the top and bottom parts are restored by the U-Net and the lipid body shapes are also restored to round shapes. Moreover, in the U-Net reconstruction, the lipid bodies indicated by the arrows are observed well while they are barely seen in the FBP reconstruction. This 3D rendering result highlights the benefit of U-Net in the 3D visualization of subcellular structures.

### 3.3. Discussion   

As a state-of-the-art method, the U-Net achieves significant improvement in image quality from the FBP reconstructions, achieving the best average RMSE value in Table 1 ▸. However, in some cases, the structures it predicts are not accurate. For example, the cell wall is not in a perfect round shape in Figs. 6 ▸(*d*) and 8 ▸(*b*). This is potentially caused by various factors such as noise, insufficient training data and over-fitting, which are ineluctable for deep learning. Because of the co-existence of the limited-angle problem and data-truncation problem in this work, where truncation is caused by the large-scale ice for immobilization of samples, applying iterative reconstruction such as simultaneous algebraic reconstruction technique with TV regularization for data consistent reconstruction (Huang *et al.*, 2019*b* ▸) to improve such incorrect structures is not feasible.

In limited angle tomography, only structures whose orientations are tangent to available X-rays can be reconstructed (Quinto, 1993 ▸, 2006 ▸, 2007 ▸; Huang *et al.*, 2016*a* ▸). Therefore, in the FBP reconstructions, most edges whose orientations are inside the scanned angular range are reconstructed. Because of this, for the chlorella reconstruction, several slices in the sagittal view contain good resolution structures. On the other hand, with the geometry setting in this work, the sagittal slices are equivalent to focus planes in tomosynthesis (Grant, 1972 ▸) where most X-rays focus. Therefore, structures viewed in sagittal planes preserve better resolution than any horizontal planes. However, structures are preserved well only in a limited number of central slices in the sagittal view, while most structures are still distorted because of missing data [Fig. 8 ▸(*a*)]. In order to view structures in any intersectional planes, artifact reduction is necessary.

Due to missing data, many essential subcellular structures are distorted or even missing in the FBP reconstruction, *e.g.* the lipid bodies in this work. The distribution and states of subcellular structures provide crucial information of intracellular activities (Ortega *et al.*, 2009 ▸; Wang *et al.*, 2015*a* ▸). With the power of deep learning in image processing, the proposed reconstruction method is competent for 3D visualization of subcellular structures, as displayed in Fig. 9 ▸. This observation indicates its important value for nanoscale imaging in biology, nanoscience and materials science.

## 4. Conclusions and outlook   

In this work, deep learning has been applied to limited angle reconstruction in TXMs for the first time. PWLS pre-processing is beneficial to improving the image quality of deep-learning reconstruction. Despite the limitation to accessing sufficient real training data, this work demonstrates that training a deep neural network model from synthetic data with proper noise modeling is a promising approach. The proposed deep-learning reconstruction method remarkably improves the 3D visualization of subcellular structures, indicating its important value for nanoscale imaging in biology, nanoscience and materials science.

Although promising and intriguing results are achieved in this work, the limited angle reconstruction problem is still not entirely resolved, since some structures are reconstructed inaccurately. In the future, the following aspects of work are worth investigating. (i) Evaluate the proposed deep-learning reconstruction method on more complex samples. (ii) More realistic noise modeling for synthetic data should potentially improve deep-learning performance. (iii) Explore new approaches to achieve data consistent reconstruction (Huang *et al.*, 2019*b* ▸) in the co-existence of the limited-angle problem and data-truncation problem. (iv) If possible, building up a database from complete real scans for training deep neural networks is necessary.

## Figures and Tables

**Table 1:** The average RMSE and SSIM values for each reconstruction method using a photon number of 10^4^ without or with PWLS

Metric	***f***_FBP_	***f***_FBP, PWLS_	***f***_U-Net_	***f***_U-Net, PWLS_
RMSE (10^−3^ µm^−1^)	2.55	2.44	1.21	1.16
SSIM	0.625	0.648	0.920	0.932
